# Diagnostic Accuracy of PSMA-PET/CT vs. mpMRI in Primary Staging of Intermediate- and High-Risk Prostate Cancer

**DOI:** 10.3390/medsci14010064

**Published:** 2026-01-31

**Authors:** Vanessa Talavera Cobo, Carlos Andres Yánez Ruiz, Mario Daniel Tapia Tapia, Andres Calva Lopez, Carmina Alejandra Muñoz Bastidas, Francisco Javer Ancizu Marckert, Marcos Torres Roca, Luis Labairu Huerta, Daniel Sanchez Zalabardo, Fernando Jose Diez-Caballero Alonso, Francisco Guillen-Grima, Jose E. Robles García, Bernardino Miñana-López

**Affiliations:** 1 Urology Department, Clínica Universidad de Navarra, 31008 Pamplona, Spain; vtalavera@unav.es (V.T.C.); cyanezruiz@unav.es (C.A.Y.R.); mdtapia@unav.es (M.D.T.T.); acalva@unav.es (A.C.L.); cmunozbasti@unav.es (C.A.M.B.); fancizu@unav.es (F.J.A.M.); mtroca@unav.es (M.T.R.); llabairu@unav.es (L.L.H.); dsanchezz@unav.es (D.S.Z.); fdcaballero@unav.es (F.J.D.-C.A.); 2 Department of Preventive Medicine, Clínica Universidad de Navarra, 31008 Pamplona, Spain; frguillen@unav.es; 3 Department of Health Sciences, Public University of Navarra, 31008 Pamplona, Spain; 4 Group of Clinical Epidemiology, Area of Epidemiology and Public Health, Healthcare Research Institute of Navarre (IdiSNA), 31008 Pamplona, Spain; 5 CIBER in Epidemiology and Public Health (CIBERESP), Institute of Health Carlos III, 46980 Madrid, Spain; 6 Urology Department, Clínica Universidad de Navarra, 28027 Madrid, Spain; bminana@unav.es

**Keywords:** PSMA PET/CT, prostate cancer, magnetic resonance imaging (MRI), staging, diagnostic accuracy, extraprostatic extension, seminal vesicle invasion, radical prostatectomy

## Abstract

Background: Prostate-specific membrane antigen (PSMA) is markedly overexpressed in prostate cancer (PCa), and there is growing evidence to support its usefulness in initial diagnostic assessments. This study compares the diagnostic performance of PSMA positron emission tomography/computed tomography (PET/CT) and magnetic resonance imaging (mpMRI) in evaluating seminal vesicle invasion (SVI), extraprostatic extension (EPE), and pelvic lymph node involvement before radical prostatectomy. Methods: A retrospective, single-institution analysis was performed. From a cohort of 325 patients who underwent radical prostatectomy between June 2022 to November 2024, 85 had undergone preoperative PSMA PET/CT for intermediate- and high-risk disease at biopsy, forming our study group. Two blinded specialists, one in radiology and one in nuclear medicine, independently interpreted the scans, using histopathological results as the reference standard. The primary outcome was diagnostic accuracy for T- and N-stage classification, while the secondary outcomes included the correct identification of the index lesion and comparative performance for each modality. Results: The study cohort comprised patients with intermediate-to-high-risk prostate cancer (median age: 66 years; median PSA level: 11.6 ng/mL; median PSA density: 0.3 ng/mL/cm^3^). Forty-eight patients presented with an ISUP grade of 3 or higher on biopsy. PSMA PET/CT was more sensitive than MRI for detecting EPE (72.2% vs. 46.9%) and nodal metastases (91.7% vs. 8.3%). Furthermore, PSMA PET/CT demonstrated significantly higher concordance with histopathological findings in index tumor localization (76.5% vs. 67.9%, *p* < 0.001). An exploratory analysis revealed a potential age-dependent pattern, but this requires confirmation in larger studies. Conclusions: In this select cohort, PSMA PET/CT demonstrated greater accuracy than MRI for locoregional staging in patients with intermediate-to-high-risk prostate cancer (PCa). However, the generalizability of these findings is limited by the retrospective design and potential selection bias. These results suggest that PSMA PET/CT may have a valuable role in the initial staging workflow, but this needs to be confirmed in larger, prospective studies. An exploratory analysis suggested a potential age-dependent pattern, but this requires confirmation in larger studies.

## 1. Introduction

Prostate cancer (PCa) is the second most common cancer in men worldwide and a leading cause of cancer incidence in Europe [1]. Accurate locoregional staging is crucial for selecting the most appropriate treatment plan, as it can prevent unnecessary extended pelvic lymph node dissections (ePLND) in low-risk patients and determine the feasibility of nerve-sparing techniques. This improves functional outcomes without compromising oncological control.

Prostate-specific membrane antigen (PSMA), a type II transmembrane protein, is overexpressed by several orders of magnitude on the surface of PCa cells compared to benign prostatic tissue [2]. PSMA positron emission tomography/computed tomography (PET/CT) utilizes this characteristic to enable the precise localization of primary and metastatic PCa foci.

Despite robust evidence demonstrating the superior accuracy of PSMA PET/CT over conventional imaging (CT and bone scans) for primary staging [3], guidelines such as those from the European Association of Urology (EAU) continue to refine its integration into clinical pathways [4]. A key unresolved question in daily practice is its performance relative to mpMRI for local staging within specific patient subgroups.

Multiparametric magnetic resonance imaging (mpMRI), which is standardized by the Prostate Imaging-Reporting and Data System (PIRADS), is the established gold standard for local staging. It provides excellent soft-tissue resolution for evaluating extraprostatic extension (EPE) and seminal vesicle invasion (SVI) [5].

However, the specific roles and comparative performance of mpMRI and PSMA PET/CT in primary staging are still being defined. Although mpMRI is highly effective in local T-staging, the ability of PSMA PET/CT to detect nodal metastases and its concordance with mpMRI in identifying the dominant intraprostatic lesion (index lesion) require further investigation in cohorts with robust histopathological correlation [6,7].

Therefore, this study aims to compare PSMA PET/CT and mpMRI head-to-head for the preoperative staging of PCa, using whole-mount histopathology as the reference standard. Specifically, we will assess their diagnostic performance in detecting EPE, SVI, and pelvic lymph node metastases, as well as in localizing the intraprostatic index lesion, and explore potential clinical predictors of imaging discordance.

## 2. Materials and Methods

### 2.1. Study Population and Patient Flow

This retrospective, single-center study identified 325 consecutive patients who underwent radical prostatectomy at Clinica Universidad de Navarra between June 2022 and November 2024. The final study cohort comprised 85 patients who had undergone a preoperative ^68^Ga-PSMA-11 PET/CT scan (Siemens Bio-graph mCT 64 and Siemens Vision 600, Siemens, Knoxville, TN, USA) as part of their clinical staging workup, forming the final study population. The decision to perform a PSMA PET/CT scan was based on institutional protocols recommending it for intermediate- and high-risk disease, or for low-risk disease with concerning features (e.g., discordant PSA kinetics or suspicious mpMRI findings). This selective referral pattern inevitably introduced spectrum bias, creating a study cohort that was enriched for higher-risk and/or MRI-suspicious disease. This issue is addressed in the Discussion section. Patients who had received any form of neoadjuvant systemic or radiation therapy before radical prostatectomy were excluded. Adjuvant therapies administered following surgery were not considered grounds for exclusion, since the histopathological analysis of the prostatectomy specimen served as the reference standard.

**Figure 1:**
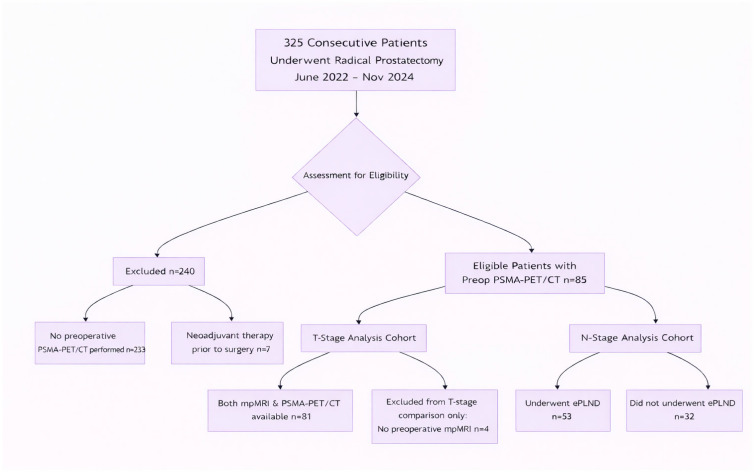
STARD Flow Diagram of Patient Selection. Abbreviations: PSMA-PET/CT: Prostate-Specific Membrane Antigen Positron Emission Tomography/Computed Tomography; mpMRI: multiparametric Magnetic Resonance Imaging; ePLND: extended Pelvic Lymph Node Dissection.

The patient selection flow is detailed in Figure 1. Of the 85 patients, 81 had both mpMRI and PSMA-PET/CT available for the primary T-stage comparison. Four patients with pT3 disease lacked preoperative mpMRI and were excluded only from the head-to-head T-stage analysis. For nodal (N-) stage analysis, the cohort consisted of the 53 patients who underwent extended pelvic lymph node dissection (ePLND).

This study was approved by the Ethics Committee of Clínica Universidad de Navarra (project number: 2025.006, Approval Date: 2 February 2025). Patient informed consent was waived since it was not required by the Ethics Committee of Clínica Universidad de Navarra, due to the retrospective nature of this study.

### 2.2. Clinical Parameters

Relevant patient information, including age, prostate-specific antigen (PSA) levels at diagnosis, and PSA density, was systematically collected. The D’Amico risk classification was calculated for each patient. The preoperative imaging assessment emphasized mpMRI and PSMA-PET/CT, and these were compared and correlated with the final histopathological findings, which are considered the gold standard. Histopathology reports from prostate biopsies were also obtained. The surgical specimens were analyzed by an expert uropathologist.

### 2.3. Definition of the Index Lesion

The histopathological index lesion was defined on whole-mount specimens after surgery by an expert uropathologist as the focus with the highest ISUP grade (the largest focus if grades were tied). For mpMRI, as the lesion with the highest PIRADS (v2.1) score, and for PSMA PET/CT, as the intraprostatic lesion with the highest maximum standardized uptake value (SUV_(max)).

A urologist then assessed topographic concordance using the same 27-sector diagram. A match was recorded if the index lesion identified through imaging (from the consensus reading) was in the same sector as the index lesion confirmed through histopathology.

### 2.4. Imaging Protocol and Analysis

1. Multiparametric MRI (mpMRI):

All patients underwent mpMRI on a 1.5-Tesla scanner (AERA, Siemens Medical Solutions, Erlangen, Germany) using a dedicated pelvic phased-array coil. Our acquisition protocol complied with PI-RADS v2.1 requirements and included: tri-planar T2-weighted turbo spin-echo sequences (slice thickness 3 mm, no gap), diffusion-weighted imaging (DWI) with b-values of 0, 100, 800, and 1400 s/mm^2^, and dynamic contrast-enhanced (DCE) imaging with temporal resolution of 12 s over 4 min following gadolinium-based contrast administration. The median time between mpMRI and PSMA-PET/CT was 28 days (IQR 14–42 days).

2. PSMA PET/CT Imaging:

Patients received a mean activity of 186.9 MBq (range: 155–215 MBq) of ^68^Ga-PSMA-11 intravenously. Imaging commenced 60 ± 10 min post-injection using a [Siemens Bio-graph mCT 64 and Siemens Vision 600, Siemens, Knoxville, TN, USA]. PET/CT system. A low-dose CT (40 mAs, 120 kVp, 2 mm slices) for attenuation correction and anatomical localization was followed by PET acquisition at 2 min per bed position. Images were reconstructed using ordered-subset expectation maximization with point-spread-function modeling (3 iterations, 21 subsets).

3. Image interpretation:

Two fellowship-trained specialists, a genitourinary radiologist and a nuclear medicine physician, with 5 and 8 years of experience, respectively, independently evaluated mpMRIs according to PI-RADS v2.1 criteria and PSMA-PET/CT scans using PSMA-RADS v1.0/PROMISE criteria. The mpMRI reads were primarily optimized for local T-staging and index lesion identification. While obvious lymphadenopathy was noted, a systematic pelvic nodal assessment with standardized size criteria was not a protocolized component of the primary readout. All reads were performed independently with readers blinded to the results of the other imaging modality and to the final histopathology. To assess the reproducibility of imaging criteria, inter-reader agreement for the key endpoints (EPE, SVI, N1 status, and index lesion localization) was calculated using Cohen’s kappa (κ) before consensus. Inter-reader agreement before consensus was substantial for PSMA-PET/CT (Cohen’s kappa = 0.78) and moderate for mpMRI (kappa = 0.62).

### 2.5. Histopathological Correlation

Whole-mount radical prostatectomy specimens that had been processed according to standard institutional protocols were used as the reference standard. All specimens were analyzed by genitourinary pathologists who were blinded to the imaging results. The analysis included tumor mapping to determine location, multifocality, and the dominant/index lesion (defined as the focus with the highest ISUP grade or the largest if grades were equal), measurement of tumor volume, assignment of ISUP grade group and assessment of surgical margin status. It also involved evaluating EPE (categorized as focal or established), SVI, and lymph node involvement (if a lymphadenectomy was performed).

#### 2.5.1. Correlation of Imaging and Histopathology

A senior urologist correlated the imaging findings with the final histopathology report. Using schematic prostate diagrams, the urologist performed a side-by-side comparison to determine topographic concordance for lesion location and extraprostatic disease. The index lesion identified on mpMRI and PSMA-PET/CT was correctly localized if it matched the histopathologically defined index lesion.

#### 2.5.2. Quality Assurance

To minimize bias, the imaging specialists were unaware of each other’s interpretations and the pathological results. All data points were cross-referenced with the electronic medical record, and any discrepancies were resolved through a consensus review process.

### 2.6. Statistical Analysis

All analyses were performed in accordance with a pre-specified statistical plan using IBM SPSS Statistics (version 29). The primary outcomes were the diagnostic accuracy of PSMA-PET/CT and mpMRI in detecting extraprostatic extension (pT3 vs. pT2) and lymph node metastasis (pN1 vs. pN0). Secondary outcomes included the detection rate of the index lesion and topographic concordance with histopathology.

We calculated the sensitivity, specificity, positive predictive value (PPV), and negative predictive value (NPV) for each modality, with 95% confidence intervals, for the primary outcomes. Accuracy, defined as the proportion of all test results that are correct (both positive and negative), was calculated using the standard formula. Diagnostic performance between modalities was compared using McNemar’s test for paired proportions.

For index lesion localization, agreement with histopathology was assessed using Cohen’s kappa. Given the well-described paradoxical effects of kappa in high-prevalence settings, results were interpreted cautiously.

Spearman’s correlation coefficients were used to evaluate the associations between PSA-related variables and PSMA-PET/CT SUV_(max). These analyses were considered exploratory and hypothesis-generating, given the non-normal distribution of the data.

For predictors of PET-positive/MRI-negative discordance, logistic regression models were fitted. The dependent variable was PET+/MRI− status (binary); independent variables included age, ISUP grade, D’Amico risk group, PSA, PSA density, and prostate volume. Model selection was based on clinical relevance and prior literature and included clinically relevant covariates: age, PSA, Gleason/ISUP group, PSA density, D’Amico risk group, and prostate volume. Due to limitations in sample size and the very low number of events (*n* = 6), only bivariable associations are reported; multivariable modeling was not feasible. Exact logistic regression was performed using the exlogistic command in Stata 18 (StataCorp LLC, College Station, TX, USA). Given the exploratory nature and statistical instability of this analysis, the full regression table is presented in Supplementary Table S1.

A two-sided *p*-value < 0.05 defined statistical significance for all pre-specified analyses.

## 3. Results

Our study population consisted of 85 patients who underwent radical prostatectomy following preoperative PSMA PET/CT scanning. Of these patients, 81 had both imaging modalities available for comparison, and 53 underwent ePLND, forming the cohort for nodal staging analysis. The cohort’s clinical and pathological characteristics are summarized in Table 1. The population was predominantly intermediate- or high-risk, with a median PSA level of 11.6 ng/mL (interquartile range (IQR): 0.6–69) and 56.5% (48 out of 85) having an International Society of Urological Pathology (ISUP) biopsy grade of at least 3. Both mpMRI and PSMA PET/CT were available for 81 patients. Final pathology confirmed extraprostatic disease (pT3) in 42.3% (36/85) of patients. Bilateral involvement was present in 54% of cases, along with EPE (28.2%) and SVI (14.1%).

When correlated with histopathology, PSMA PET/CT showed significantly better tumor lateralization (Cohen’s kappa = 0.7) and index lesion detection (76.5% accuracy) than mpMRI (kappa = 0.4; 67.9% accuracy; both *p* < 0.001).

### 3.1. T-Stage

For detecting extraprostatic extension (T3 stage), PSMA PET/CT showed a sensitivity of 72.2% (26/36), compared to 46.9% (15/32) for mpMRI (*p* = 0.02). The respective specificities were 79.6% (39/49) and 81.6% (40/49) (Table 2). The negative predictive value (NPV) of PSMA PET/CT for ruling out EPE was 79.6% (39/49), compared to 70.2% (40/57) for mpMR. Four patients with pT3 disease did not have MRI data and were excluded from this analysis. This non-random missing data may lead to an overestimation of mpMRI’s diagnostic performance in our cohort.

The mean SUV_(max) in index lesions was 13.87. Spearman’s correlation analysis revealed a statistically significant, albeit modest, positive correlation between diagnostic PSA levels and lesion SUV_(max) (ρ = 0.352, *p* < 0.001). This modest correlation suggests that PSA and PSMA uptake are related. However, correlation analysis alone cannot be used to infer predictive value. Therefore, no causal interpretation should be drawn, and regression-based approaches would be required to assess independent predictive ability.

### 3.2. N-Stage

Of the 53 patients who underwent ePLND, 12 (22.6%) had lymph node metastases (pN1). In this cohort, PSMA PET/CT demonstrated a sensitivity of 91.7% (11/12) and an NPV of 97.6% (40/41), substantially higher than mpMRI, which had a sensitivity of 8.3% (1/12) and an NPV of 80.5% (33/41) (Table 2). The accuracy of PSMA PET/CT for nodal staging was 96.2% (51/53) compared to 79.3% (42/53) for mpMRI (*p* = 0.01 by McNemar’s test).

Although the nodal dissection templates included the obturator, external iliac, and internal iliac regions, a detailed, region-by-region pathological correlation was unavailable. Therefore, it was not possible to evaluate PET–pathology concordance for specific nodal stations directly. Both modalities showed high specificity (PSMA PET/CT: 97.6%; MRI: 97.8%; see Table 2). Notably, PSMA PET/CT detected an atypical metastatic lesion that was histologically confirmed following targeted resection. The median short-axis diameter of PSMA PET/CT-detected lymph node metastases was 8.5 mm (range 4–15 mm).

### 3.3. Exploratory Analysis of Imaging Discordance

An exploratory, descriptive analysis was performed on 7.1% of cases (6/85) with PET-positive/MRI-negative discordance, and exact logistic regression was employed. Due to the very small number of events, formal inferential modeling was not pursued to avoid overfitting, and the results should be interpreted with extreme caution as hypothesis-generating only. The full results are presented in Supplementary Table S1.

A post hoc analysis suggested that patients aged ≤ 62 years had higher odds of discordance (OR = 8.24, 95% CI: 1.07–99.05; *p* < 0.05). However, this model is severely unstable due to the very small number of discordant cases (*n* = 6), as reflected by the extremely wide confidence interval, and the threshold was derived from the data. Therefore, this finding is preliminary and requires validation in a larger, adequately power prospective cohort.

## 4. Discussion

In this cohort of intermediate-to-high-risk prostate cancer patients, PSMA PET/CT was found to play a clinically complementary role to mpMRI. It was found to provide superior accuracy for nodal staging in this real-world setting. Our data support integrating PSMA PET/CT into the primary staging workflow to refine risk assessment and guide treatment planning, while confirming mpMRI’s central role in local assessment.

Since its introduction to the clinic in 2012, PSMA PET/CT has become a key imaging modality for PCa, particularly for detecting biochemical recurrence [6]. Its diagnostic performance is influenced by the choice of radioligand (e.g., ^68^Ga or ^18^F) and correlates with PSMA uptake intensity. The intensity of PSMA uptake, quantified by SUVmax, is known to correlate with disease aggressiveness [8,9].

The sensitivity of PSMA PET/CT shows a strong positive correlation with disease aggressiveness, with progressively higher detection rates at increasing PSA levels and higher Gleason scores [7,10,11]. This association highlights its value in patients with intermediate-to-high-risk disease, in whom PSMA PET/CT achieves a sensitivity of 97% for PSA > 10 ng/mL and GS ≥ 8 [7,11]. This is superior to MRI, which achieves a sensitivity of 87% [5]. However, its performance is limited in low-risk disease, providing meaningful benefits mainly when PSA exceeds 9.4 ng/mL, and the patient is over 62.5 years old [5]. A meta-analysis supports these observations further, reporting per-patient and per-lesion sensitivities of 93.4% and 81.6%, respectively [2].

Our analysis confirms the distinct yet complementary strengths of PSMA PET/CT and mpMRI. For local tumor assessment, our findings are consistent with those of prospective trials, which highlight the superiority of mpMRI in detecting EPE and SVI [12]. Conversely, PSMA PET/CT demonstrated greater diagnostic accuracy for nodal metastasis in our cohort (96.2% versus 79.3% for MRI; an increase of 16.9 percentage points). This performance is consistent with studies emphasizing its high specificity [13,14,15,16] and supports its use in decision-making regarding ePLND [17,18,19]. This bridges the gap between European [4] and NCCN [20] risk thresholds. Therefore, our results support a complementary, rather than substitutive, role for PSMA PET/CT in preoperative staging [21].

The diagnostic performance of PSMA PET/CT observed in our study must be contextualized within a rapidly evolving landscape. Although our nodal sensitivity was higher than the 40% reported in some earlier studies [22], this is probably due to the risk profile of our cohort and improvements in imaging protocols. Landmark trials such as ProPSMA [23] and PRIMARY [24] have robustly demonstrated that PSMA PET/CT significantly improves staging accuracy, particularly when combined with MRI. Integrating standardized reporting systems such as the PRIMARY score, which our study utilized, reduces interpretive variability and enhances local disease assessment [25,26,27,28,29,30].

An exploratory analysis suggested a potential inverse relationship between patient age and the diagnostic yield of PSMA PET/CT. We found that the likelihood of imaging discordance decreased by 9% for every additional year of age (OR = 0.91/year), indicating substantially greater detection capability in patients aged 62 years or younger. This effect may be driven by three factors: (1) a biological predisposition to more aggressive, PSMA-avid tumors in younger patients, (2) technical advantages, such as improved contrast resolution in smaller prostate volumes, and (3) the limitations of mpMRI in evaluating the transitional zone. zone. It is critical to emphasize that this finding is based on only six discordant cases, resulting in severe statistical instability (wide CI: 1.07–99.05). Therefore, it must be considered a strictly hypothesis-generating approach and requires validation in a large, prospective cohort before any clinical implications can be drawn.

### Limitations

Several limitations must be considered when interpreting our findings. The most significant of these is the spectrum bias resulting from non-random referral for PSMA-PET/CT, which enriches the cohort with higher-risk disease. This likely overestimates the modality’s sensitivity and accuracy relative to an unselected population and affects the mpMRI comparison. Further limitations include the retrospective, single-center design, which may introduce selection and information biases. Furthermore, while the sequential imaging protocol (PSMA PET/CT followed by MRI) reflects real-world clinical practice, it could have influenced interpretation. Other limitations include the modest sample size (*n* = 85), which limited subgroup analyses, and the fact that mpMRI was optimized for local staging, which undermined the head-to-head N-stage comparison. Finally, the lack of region-by-region correlation for lymph node metastases precludes a more granular analysis of PET/CT performance. These clinically plausible patterns underscore the need for validation in larger, less selected, prospective cohorts.

In conclusion, our data reinforce the idea that PSMA PET/CT and mpMRI are synergistic, not competing, modalities in the pre-operative staging of intermediate-to-high-risk prostate cancer. mpMRI remains the cornerstone for evaluating local disease and guiding nerve-sparing surgery, while PSMA PET/CT provides indispensable information for nodal and systemic staging. The potential for enhanced utility in younger patients could pave the way for more personalized, cost-effective imaging algorithms.

## 5. Conclusions

PSMA PET/CT offers more accurate nodal staging than mpMRI in this selected group of patients. An exploratory analysis suggested that it may be more valuable in younger patients, but this requires rigorous prospective validation. Further research is required to develop risk-adapted imaging algorithms that consider clinical factors such as age.

## Figures and Tables

**Table 1:** Baseline Clinical, Imaging, and Pathological Characteristics of the Study Cohort.

	*n* = 85
Age, years, median (IQR)	66 (47–79)
Initial PSA level, ng/mL, median (IQR)	11.6 (0.6-69)
Initial PSA Density, ng/mL/cm^3^, median (IQR)	0.3 (0.03–1)
D’Amico risk classification, *n* (%)	
Low risk	3 (3.5)
Intermediate risk	50 (58.8)
High risk	32 (37.6)
ISUP of prostate biopsy > 3, *n* (%)	48 (56.5)
Prostate biopsy laterality, *n* (%)	
Right	18 (21.2)
Left	28 (32.9)
Bilateral	39 (45.9)
mpMRI findings (patients)	
No MRI (4)	4
No suspicious lesion	11
PIRADS 3	3
PIRADS 4	31
PIRADS 5	36
PSMA PET/CT PROMISE miTNM score (patients)	
miT2	49
miT3a	21
miT3b	15
miN1	15
PSMA PET/CT SUV max index lesion, median (IQR)	13.9 (2–88)
Final ISUP grade, *n* (%)	
ISUP 1	9 (10.6)
ISUP 2	38 (44.7)
ISUP 3	24 (28.2)
ISUP 4	4 (4.7)
ISUP 5	10 (11.8)
Final Pathological T stage, (patients, %)	
pT2	49 (57.6)
pT3a	24 (28.2)
pT3b	12 (14.1)
Final Pathological N stage (patients, *n* = 53)	
pN0	41
pN1	12

**Table 2:** Diagnostic accuracy of MRI and PSMA PET/CT scans (Data % (95% CI)).

	T3 STAGE		N1 STAGE	
	PSMA PET/CT	mpMRI	PSMA PET/CT	mpMRI
Sensitivity	72.2 (56–84)	46.9 (30.9–63.5)	91.7 (61.5–99.8)	8.3 (0.2–38)
Specificity	79.6 (66.3–88.5)	81.6 (68.6–90.0)	97.6 (87.4–99.9)	97.8 (88–99.8)
PPV	72.2 (56.1–84.1)	62.5 (42.7–78.4)	91.7 (61.5–99.8)	50% (1.3–98.7)
NPV	79.6 (66.36–88.5)	70.2 (57.3–80.5)	97.6 (87.4–99.9)	80.5 (70–88)
Diagnostic Accuracy	76.5 (66.4–84.2)	67.9 (57.1–77.1)	96.2 (86.5–99.5)	79.3 (68–87)
Cohen’s Kappa	0.51 (0.31–0.73)	0.29 (0.09–0.51)	0.89 (0.74–0.99)	0.09 (−0.37–0.55)

## Data Availability

The original contributions presented in this study are included in the article. Further inquiries can be directed to the corresponding author.

## Supplementary Materials

The following supporting information can be downloaded at: https://www.mdpi.com/article/10.3390/medsci14010064/s1, Supplementary Table S1. Exact logistic regression results (one-sided test) for predictors of PET-positive/MRI-negative discordance in cases of histologically confirmed prostate cancer (*n* = 85, with 6 discordant cases).

## Abbreviations

The following abbreviations are used in this manuscript:

CI	Confidence interval
DCE	Dynamic Contrast-Enhanced
DWI	Diffusion-Weighted Imaging
EPE	Extraprostatic Extension
ePLND	Extended Pelvic Lymph Node Dissection
IQR	Interquartile Range
ISUP	International Society of Urological Pathology
miTNM	Molecular imaging TNM
mpMRI	Multiparametric Magnetic Resonance Imaging
NPV	Negative Predictive Value
OR	Odds ratio
PCa	Prostate Cancer
PET/CT	Positron Emission Tomography/Computed Tomography
PI-RADS	Prostate Imaging-Reporting and Data System
PPV	Positive Predictive Value
PSA	Prostate-Specific Antigen
PSMA	Prostate-Specific Membrane Antigen
STARD	Standards for Reporting Diagnostic Accuracy Studies
SVI	Seminal Vesicle Invasion
SUVmax	Maximum Standardized Uptake Value
TNM	Tumor–Node–Metastasis staging system
