# Supplementary material for: Diagnostic Accuracy of PSMA-PET/CT vs. mpMRI in Primary Staging of Intermediate- and High-Risk Prostate Cancer
Source: Med Sci (Basel). 2026 Jan 31;14(1):64. doi: 10.3390/medsci14010064 (PMC12921803; doi:10.3390/medsci14010064)
Supplement: Supplementary file 1 [file medsci-14-00064-s001.zip › medsci-4049071-supplementary.pdf]

medical sciences

MDPI

Table S1. Exact logistic regression results (one-sided test) for predictors of PET-positive/MRI-negative discordance in cases of histologically confirmed prostate cancer (N=85, with 6 discordant cases).

Predictor	Odds Ratio	95% CI	p-value
Age (continuous)	0.9	0.81-1.02	0.04
ISUP grade (ordinal, 1-5)	1.7	0.82-3.94	0.17
ISUP grade (binary:3-5 vs 1-2)	4.1	0.43-203.38	0.20
D'Amico risk group (ordinal, 1-5)	1.1	0.94-1.45	0.10
D'Amico risk group (binary:3-5 vs 1-2)	--	Not estimable	--

Note: Due to the very small number of events (n=6), these results are statistically unstable and should be interpreted as strictly hypothesis-generating. CI: Confidence Interval.
